# Design Analysis for Controlling Spray Particle Size of Ultrasonic Nozzles Using Piezoelectric Ceramic Vibrators

**DOI:** 10.3390/ma19112245

**Published:** 2026-05-26

**Authors:** Su-Ho Lee, Sunghyun Lim, Myeong-Gwang Choi, Jae-Eun Hwang, Herie Park

**Affiliations:** 1 Department of Electrical Engineering, Dong-A University, Busan 49315, Republic of Korea; leesuho@dau.ac.kr (S.-H.L.); dlatjdgus955@gmail.com (S.L.); see0070@nate.com (M.-G.C.); wjsdmlwh@naver.com (J.-E.H.); 2 Department of ICT Integrated Safe Ocean Smart Cities Engineering, Dong-A University, Busan 49315, Republic of Korea

**Keywords:** ultrasonic nozzle, high Curie temperature, spray particle, COMSOL analysis, theoretical analysis of spraying

## Abstract

This study aims to demonstrate the feasibility of controlling particle size through a mathematical model in the design of industrially applicable ultrasonic spray nozzles by utilizing the vibrational characteristics of piezoelectric ceramics. A piezoelectric ceramic composition with a low sintering temperature and excellent thermal stability (Curie temperature above 300 °C) was developed and used as a ceramic vibrator. Furthermore, the resonance frequency and nozzle displacement were calculated using the COMSOL program and applied to a mathematical model to design an ultrasonic nozzle capable of producing a spray particle diameter of approximately 30 μm. The designed ultrasonic nozzle was fabricated, and its spray characteristics were analyzed. The consistency of the spray characteristics was examined by comparing them with the mathematical model based on changes in ultrasonic nozzle length, resonance frequency, and fluid viscosity. When the ultrasonic nozzle horn length was 22 mm, the resonance frequency was found to be 42.1 kHz, and at a flow rate of 65 mL/min. the average spray particle size was approximately 30–40 μm, indicating fine and uniform particles. In addition, it can be seen that as the length of the nozzle horn increases, the resonance frequency decreases, reducing the supply energy delivered to the liquid, and the particle size increases as shown in the mathematical analysis. The theoretical separation energy required to atomize pure water at a flow rate of 65 mL/min. is 2100 J, which was found to be greater than all energy loss occurring during the atomization process. However, it can be seen that as the length of the ultrasonic nozzle increases, the maximum atomization volume increases, and as viscosity increases, the energy required to separate a single atomized particle becomes greater.

## 1. Introduction

Currently, research is being conducted on various sensors utilizing the direct piezoelectric effect of piezoelectric ceramics, as well as on piezoelectric harvesting devices in the renewable energy sector [1,2,3,4]. Furthermore, the development of various application devices utilizing the inverse piezoelectric effect to obtain mechanical energy is actively being studied in industrial settings [5,6,7,8,9,10]. In particular, ultrasonic vibration energy utilizes the inverse piezoelectric effect of piezoelectric ceramics, which generates significant mechanical force and thus generates high output energy. The most common method for atomizing flowing liquids is to create a hole in the tip, compress the liquid, and then spray it [11]. A typical method involves controlling the size of the hole and the pressure applied to the liquid to control the spray droplet size. However, in this method, in order to reduce the particle size of the liquid to be sprayed, the size of the nozzle must be reduced, which often leads to accidents where the nozzle becomes clogged by foreign substances, etc., and there is a problem that the particle size of the sprayed liquid is not uniform. However, in the case of nozzles that use ultrasonic energy, the nozzle is large, and the particle size can be adjusted uniformly according to the ultrasonic frequency, so this problem can be solved. In addition, an ultrasonic humidifier is a representative product that generates a general ultrasonic spray. This method generates a spray by attaching an ultrasonic vibrator to the center of a still fluid and transmitting ultrasonic mechanical vibration energy to the center of the fluid, and a constant water level must always be maintained. Therefore, devices that spray using a method such as an ultrasonic humidifier have the disadvantage of being very difficult to use by finely atomizing liquid fuel flowing in a pipe. However, the ultrasonic nozzle proposed in this study can be installed in the middle of a pipe through which a fluid passes and sprays the fluid coming out of the outlet directly, making it useful as a spray device for liquids flowing in a pipe. Therefore, in this study, we presented a theory of spraying ultrasonic nozzles that can generate sprays from flowing fluids and developed a model that can obtain maximum displacement characteristics using the COMSOL program(Multiphysics and Structural Mechanics Module and AC/DC Module version 6.3). We selected a model that produces maximum displacement. We also compared and reviewed it with the model created by manufacturing an actual ultrasonic nozzle. Furthermore, to examine the possibility of controlling the spray particle size, we examined the spray characteristics by changing the type of fluid and the change in spray particles by changing the flow rate (flow rate). In addition, we supported the theory by comparing the results of theoretically obtaining the amount of energy used to stabilize the ultrasonic nozzle with the energy used in an actual ultrasonic nozzle [12,13,14]. This study aims to design an ultrasonic nozzle that controls the uniform atomization particle size of diesel fuel particles in order to develop a high-efficiency industrial combustion boiler capable of contributing to the resolution of recent environmental and energy issues.

## 2. Ultrasonic Nozzle Manufacturing and Atomization Theory

### 2.1. Piezoelectric Ceramic Manufacturing

Perovskite-type piezoelectric ceramics possess an ABO_3_ crystal structure and exhibit excellent characteristics for converting electrical energy into mechanical energy. While PZT-based ceramics have been the primary focus of research to date, studies aimed at improving properties by substituting various materials into the B-site have recently been conducted. Therefore, in this study, devices were fabricated by substituting (Mn_1_/_3_Sb_2_/_3_) into the B-site to enhance the sinterability of the ceramic. However, the purpose of this study was not to develop a ceramic vibrator with superior physical and electrical properties. However, the goal was to utilize the superior vibration efficiency of the ceramic vibrator to enhance efficiency through fine atomization of liquid fuel, thereby contributing to environmental issues. Therefore, since ceramic manufacturing typically requires sintering at high temperatures, typically above 1200 °C, the substitution amount of PMS was varied to minimize the volatilization of Pb during sintering [15,16,17,18,19,20]. A two-stage calcination process was used: a first calcination at 700 °C for 4 h and a second calcination at 750 °C for 2 h [21]. The sintering temperature was then lowered to around 900 °C [22]. The composition of the device used in this study was selected for manufacturing, with a PMS(Pb(Mn_1_/_3_Sb_2_/_3_)O_3_) substitution ratio of 3 mol%, which exhibits relatively excellent electrical properties. The compositional formula for the device used in this study is as follows [23,24].

Pb(Mn_1/3_Sb_2/3_)_x_(Ni_1/3_Nb_2/3_)_0.14−x_(Zr_1/2_.Ti_1/2_)_0.86_O_3_ + 0.1 wt.% MnO_2_ + 0.2 wt.% Fe_2_O_3_ + 0.2 wt.% CuO (x = 0, 0.03, 0.05, 0.07, 0.09 mol)

**Figure 1:**
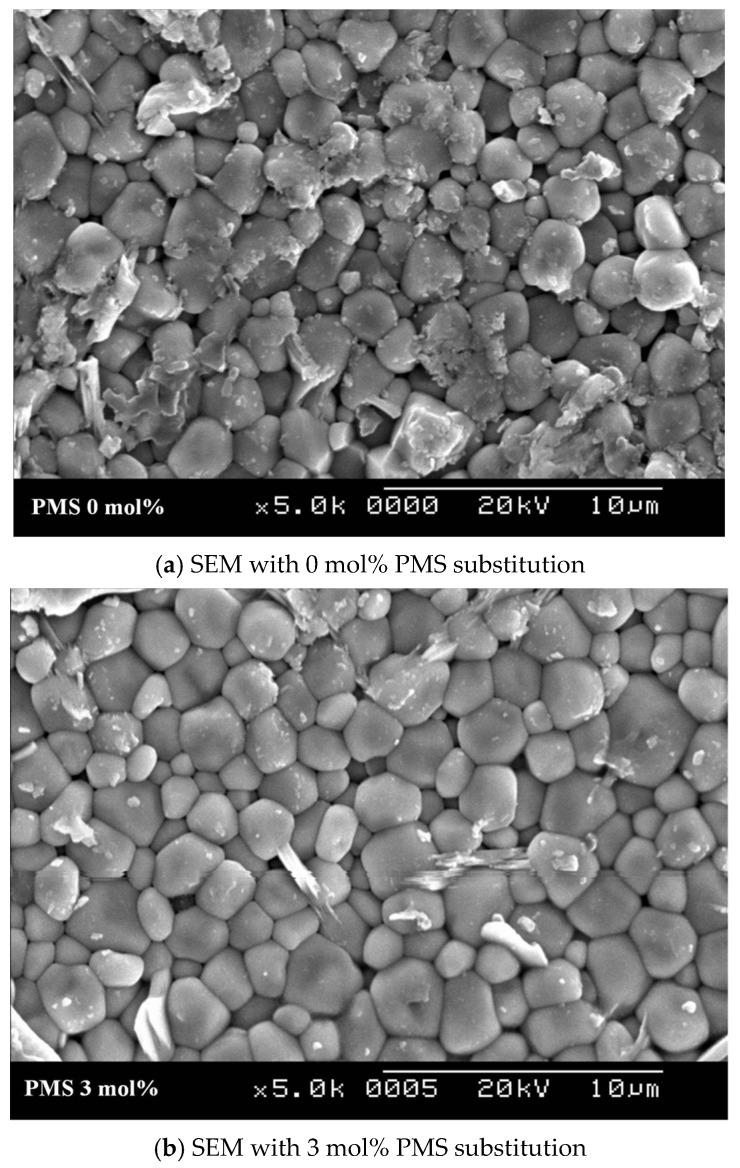

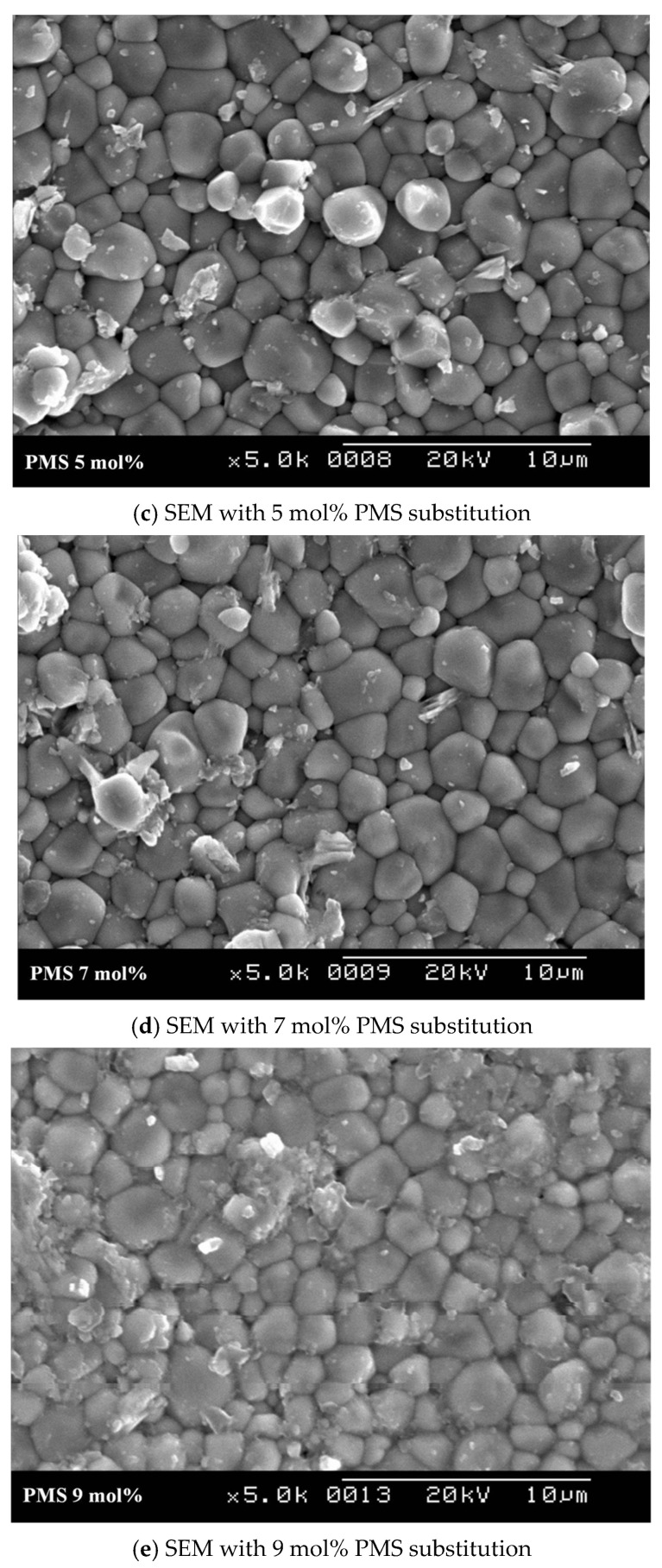
SEM images according to PMS substitution amount.

Figure 1 shows the microstructure of the specimens according to PMS substitution using SEM(JSM-6700F, JEOL, Tokyo, Japan). It can be seen that the uniformity of the specimen’s particle size increased with increasing particle size at a PMS substitution amount of 3 mol%. However, beyond that, the non-uniformity of the particle size increased. Therefore, it is thought that excellent electrical properties and sinterability will increase at a PMS substitution amount of 3 mol%.

**Figure 2:**
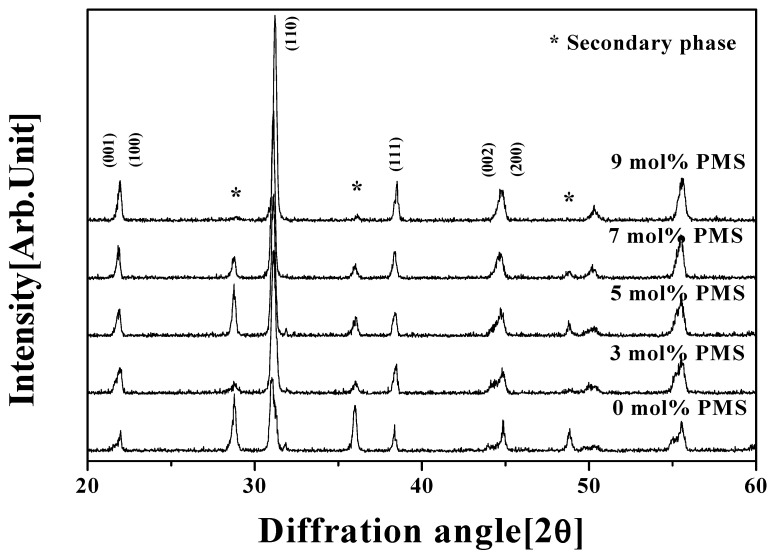
Analysis of XRD patterns according to PMS substitution amount.

Figure 2 shows the XRD(EMPYREAN, Malvern, Worcestershire, UK) patterns of the specimens according to PMS substitution. Although secondary phases appeared in all specimens, the intensity of the secondary phases tended to decrease as the amount of PMS increased. This phenomenon is considered to be decisive evidence that PMS substitution improves sinterability in this composition. Furthermore, as the amount of PMS substitution increased, the crystal structure transitioned from a tetragonal to a rhombohedral phase, and since a phase boundary region was observed when the amount of PMS substitution was 3 mol%, it was expected that high electrical properties could be obtained.

**Figure 3:**
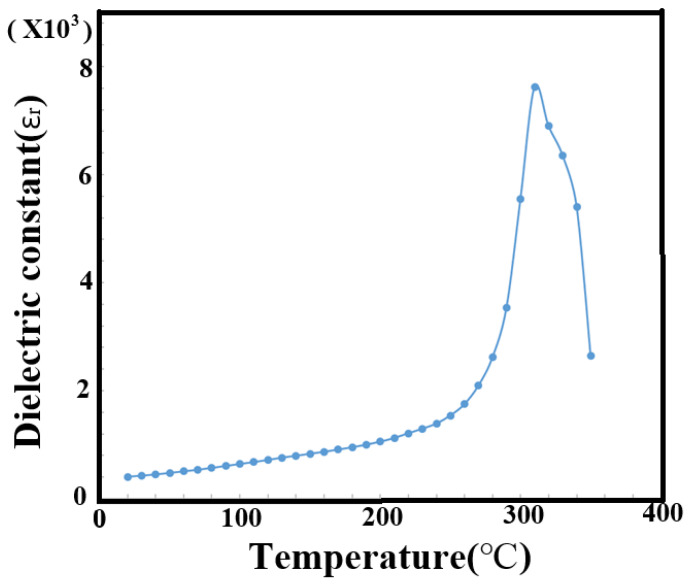
Temperature-dependent dielectric constant of piezoelectric ceramics substituted with 3 mol% of PMS.

In the case of ultrasonic transducers, since they generate mechanical vibrations, the higher the Curie temperature of the ultrasonic ceramic, the better, in order to increase stability due to heat generation. Therefore, the Curie temperature of the piezoelectric ceramic used in this study was confirmed and is shown in Figure 3. When the substitution amount of PMS was 3 mol%, it was expected to exhibit a high Curie temperature of approximately 310 °C, thereby ensuring temperature stability.

Table 1 summarizes the electrical characteristics of the ceramic used as an ultrasonic vibrator in this study. For investigating the dielectric properties, capacitance was measured at 1 kHz using an LCR meter (ANDO AG-4034, Hioki, Ueda, Japan, 2025) and ε_r_ was calculated. For investigating the piezoelectric properties, the resonant and antiresonant frequencies were measured by an Impedance Analyzer (4294A, Agilent, Santa Clara, CA, USA, 2003) according to IEEE standard and then the k_p_ and Q_m_ were calculated. And the piezoelectric constant (d_33_) was measured using a d_33_ Meter (YE2730A, Sinocera, Shandong, China, 2024). It was found that when 3 mol% of PMS was added, the piezoelectric and electrical characteristics were superior compared to other addition amounts. Additionally, it was found that the overall characteristics were not inferior when compared to PZT-based ceramics reported in various papers shown in Table 1 [25,26,27]. Therefore, a ceramic vibrator was fabricated using a piezoelectric ceramic with 3 mol% of PMS substituted and used as a vibrator for an ultrasonic nozzle.

### 2.2. Fabrication of Ultrasonic Nozzles

**Figure 4:**
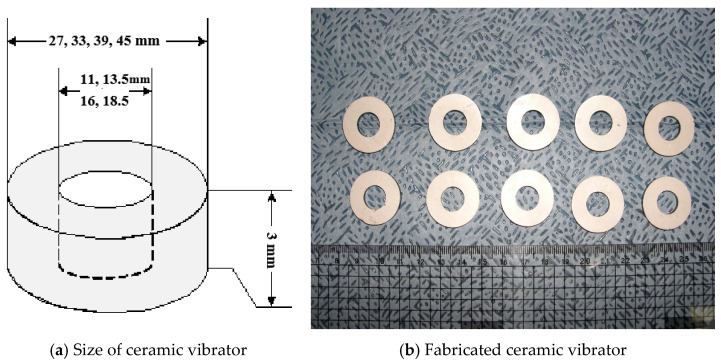
Piezoelectric ceramic vibrator.

To examine the atomization characteristics of ultrasonic nozzles according to their length, the ultrasonic nozzle vibrators for this study were manufactured using CIP (Cold Isostatic Press: Kintek, China, 2009) at 19,500 psi and sintered at 900 °C. Figure 4a shows the vibrator size according to the nozzle size, and Figure 4b shows the sintered specimens [26].

**Figure 5:**
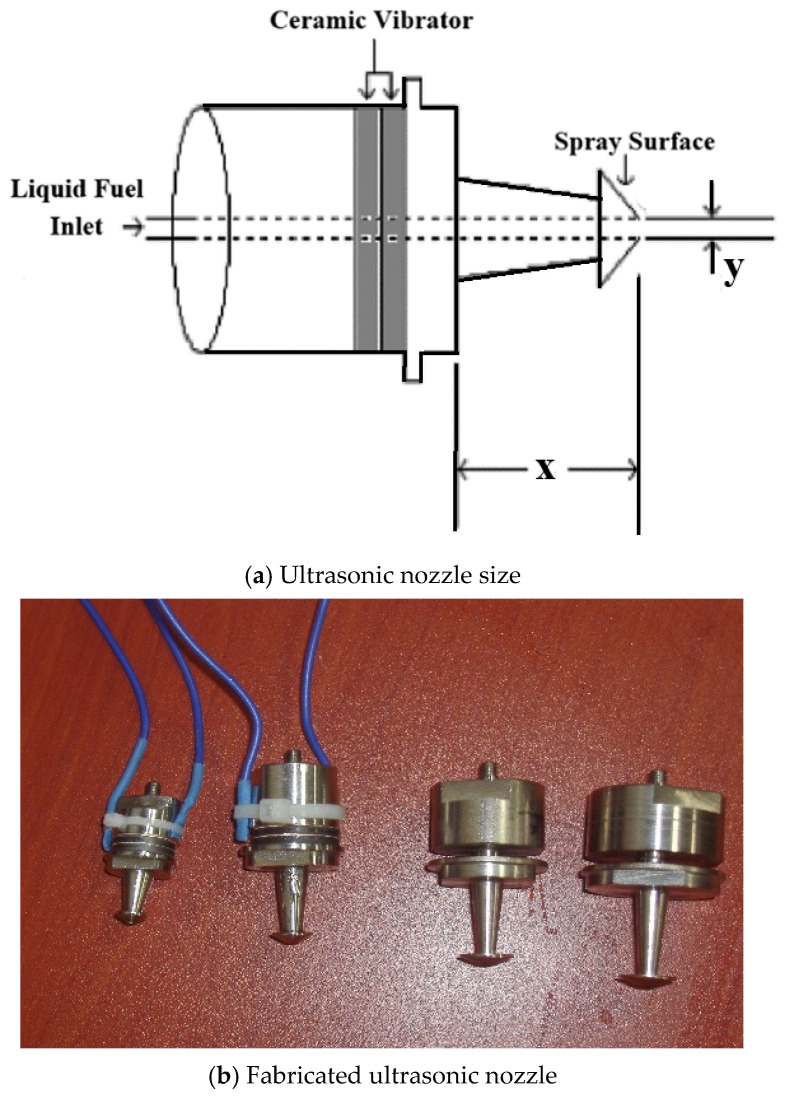
Configuration of the ultrasonic nozzle.

In this study, the ultrasonic nozzle body was tested using steel, brass, and SUS304 materials. However, brass exhibited an elastic modulus approximately half that of steel and SUS304, resulting in no atomization; conversely, steel was found to be unsuitable as a material for the nozzle body due to surface oxidation. Therefore, the nozzle body was fabricated using SUS304, a type of stainless steel. In addition, to increase the generation output of ultrasonic waves, two ceramic vibrators were combined and connected in parallel as shown in Figure 5a. In addition, in order to compare the characteristics of the amount of liquid sprayed and the size of the sprayed particles, the length x of the nozzle horn through which the vibration energy is transmitted was changed to 22, 27, 32, and 37 mm and manufactured. In addition, in order to examine the characteristics of the size of the sprayed particles and the spray distance according to the change in the flow rate passing through the nozzle pipe, the size y of the fluid inlet was manufactured to be 1.8 mm. Figure 5b shows a photograph of the actually manufactured ultrasonic nozzle.

### 2.3. Theoretical Analysis of Spraying from an Ultrasonic Nozzle

The atomization phenomenon of an ultrasonic nozzle can be explained by understanding the energy flow received by the liquid from the time it is injected into the nozzle until it reaches the atomizing surface. The kinetic energy of a moving object is generally expressed as follows:

(1) $$\epsilon =\frac{1}{2}mv^{2}+V$$

Here, *m* is the mass of the moving object and *v* is the velocity of the object. In Equation (1), the first term on the right-hand side is the kinetic energy of the object, and *V* is the potential energy of the object. For the convenience of theoretical interpretation, it is assumed that the potential energy is 0, so only the kinetic energy of the object exists. First, in order to interpret the spraying phenomenon mathematically, we can first consider that when the ceramic vibrator vibrates once, the injected fluid particle collides once with the nozzle tube wall. First, to obtain the energy received by a particle during a single collision based on the mass and collision time of the particle, we introduce momentum *P* = *mv* and substitute it into Equation (1). Additionally, by applying the fact that the change in momentum is impulse, we can set the impulse received by a single particle as *F =* ∆*P* = *dP*/*dt*. Therefore, by integrating both sides of *Fdt = dP*, we obtain *F·τ = P*, which yields Equation (2) below. Here, *τ* represents the time taken for a single liquid particle to collide.

(2) $$∆\epsilon =\frac{{∆P}^{2}}{2m}=\frac{{\left(F\tau \right)}^{2}}{2m}$$

In Equation (2), m represents the mass of one particle, F represents the force applied to one particle, and τ represents the collision time of one particle. Here, when a piezoelectric ceramic vibrator is used as an ultrasonic element, the force intensity F generated and the collision time τ must be rearranged using the piezoelectric constant of the ceramic. In general, the force Fc that causes the vibration of a ceramic vibrator can be rearranged as in Equation (3).

(3) $$F_{c}=\frac{P}{A}=\frac{DV}{A}=m\cdot a$$

In Equation (3), A represents the cross-sectional area of the ceramic vibrator, D is the shape ratio of the ceramic vibrator (the ratio of the thickness and cross-sectional area of the ceramic vibrator X the piezoelectric charge constant (g)), *V* represents the applied voltage and *a* represents acceleration. In addition, Equation (3) can be rearranged as in Equation (4) to obtain the collision time τ.

(4) $$\tau^{2}=\frac{2r}{a}=\frac{2r\cdot m}{F_{c}}$$

In Equation (4), *r* represents the radius of the liquid particle. By substituting τ obtained from Equation (4) into Equation (2), we can obtain the amount of energy obtained by a single particle of the fluid injected into the nozzle in a single collision, as in Equation (5).

(5) $${∆\epsilon }_{1}=\frac{F_{c}^{2}}{2m}\frac{2r\cdot m}{F_{c}}=F_{c}\cdot r$$

By substituting the force *Fc* of the ceramic vibrator obtained from Equation (3) into Equation (5), the energy received by one liquid particle per vibration can be obtained using Equation (6).

(6) $${∆\epsilon }_{1}=\frac{D\cdot rV}{A}$$

However, the total number of collisions that occur when one liquid particle passes through the nozzle tube can be expressed by considering the length of the nozzle tube, the ceramic vibration frequency, and the velocity of the fluid, as in Equation (7). In this case, it was assumed that the liquid particle collides twice for every one vibration frequency.

(7) $$N=\frac{2l\cdot f}{v_{l}}$$

Here, *f* is the vibration frequency of the ceramic, and *l* is the length of the nozzle tube. Additionally, *v_l_* represents the fluid flow velocity through the tube. Therefore, the total energy that a single liquid particle can obtain as it passes through the tube is as shown in Equation (8).

(8) $${∆\epsilon }_{N}=\frac{2D\cdot rV\cdot l\cdot f}{Av_{l}}$$

Here, Equation (7) represents the case where 100% of the collision energy is transferred to the liquid as many times as the vibration frequency in the ideal case where no loss is considered in the liquid particles. However, the viscosity of the liquid and the liquid in motion play a role in reducing the vibration energy generated by the collision. Therefore, by taking this into account in Equation (8) and taking into account the energy reduction due to viscosity and the motion of the liquid, the energy actually received by one liquid particle can be expressed as in Equation (9).

(9) $$∆E=\frac{2D\cdot rV\cdot l\cdot f}{Av_{l}}-\frac{1}{2}mv_{l}^{2}(1-e^{-\frac{tl}{\tau }})$$

Here, tl/τ can be used as the coefficient of viscous friction η that a liquid spherical particle with viscosity experiences when moving, as a relationship between the density and viscosity of the fluid, and can be changed to $\tau =\left(\frac{b}{m}\right),\;tl=(\frac{l}{v_{l}})$. Here, b represents the damping constant, and m represents the mass. The exponential part of the last term on the right side of Equation (9) can be rearranged as in Equation (10).

(10) $$\frac{tl}{\tau }=\frac{m\cdot l}{bv_{l}}=\frac{\frac{4}{3}\pi r^{3}\rho l}{6\pi \eta r\cdot v_{l}}=\frac{2lr^{2}\rho }{9\eta v_{l}}$$

Here, η is the viscosity coefficient of the liquid, and ρ is the density of the liquid. Therefore, by substituting Equation (10) into Equation (9) and rearranging it, we can see that Equation (11) is obtained. Therefore, the energy transferred to a single particle must be greater than the sum of the surface tension energy of the viscous liquid, the heat energy of the ultrasonic nozzle, the energy converted into sound, and other loss energies (Electrical loss, load loss, etc.) to exhibit the spray phenomenon in which the particles are separated.

(11) $$\Delta E=\frac{2D\cdot rV\cdot l\cdot f}{Av_{l}}-\frac{1}{2}mv_{l}^{2}(1-e^{-\frac{2lr^{2}\rho }{9\eta v_{l}}})$$

According to Equation (11), it can be seen that the increase in energy received by a single liquid particle is greatly affected by the resonant frequency. Therefore, in order to change the resonant frequency, it is necessary to change the length of the sprayer. Therefore, the length of the horn of the sprayer is changed to 22 mm, 27 mm, 32 mm, and 37 mm, and the spraying characteristics are experimentally examined to show that they are in good agreement with the theoretical analysis [28,29].

## 3. Results and Discussion

**Figure 6:**
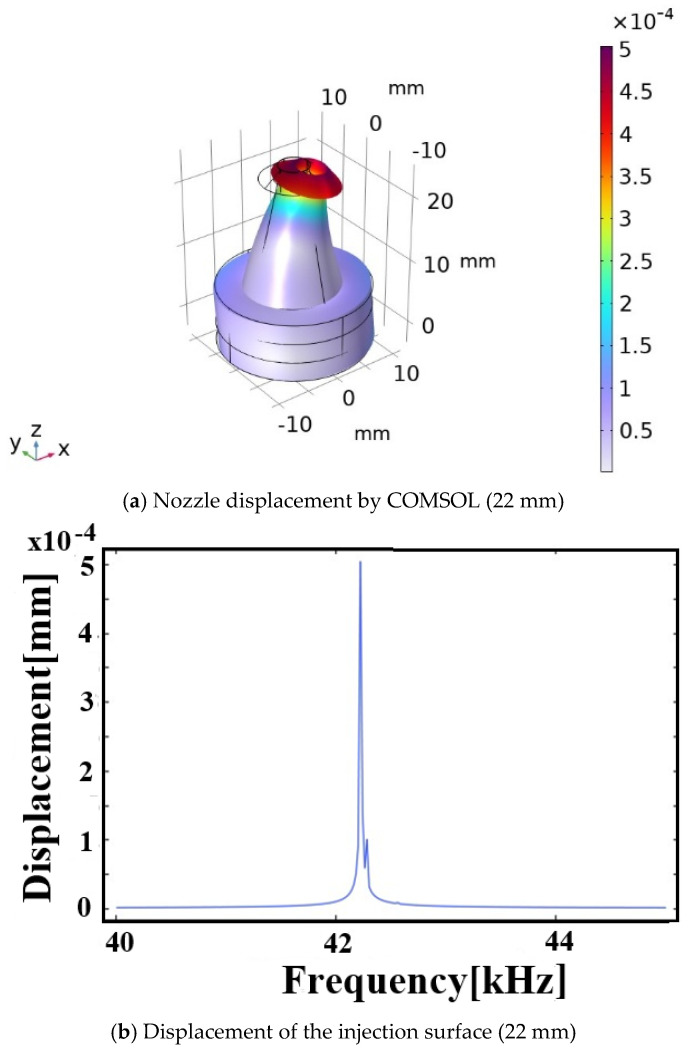

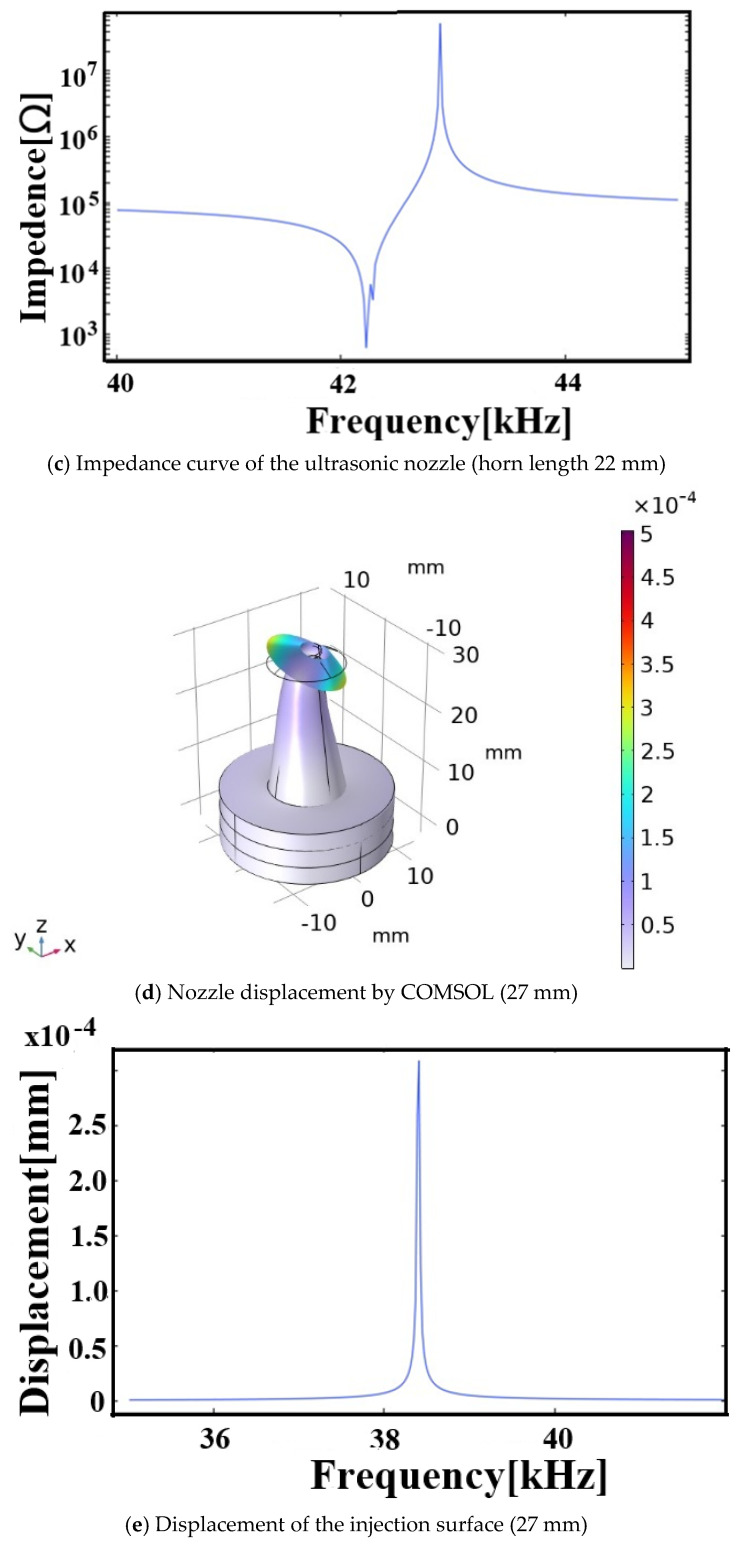

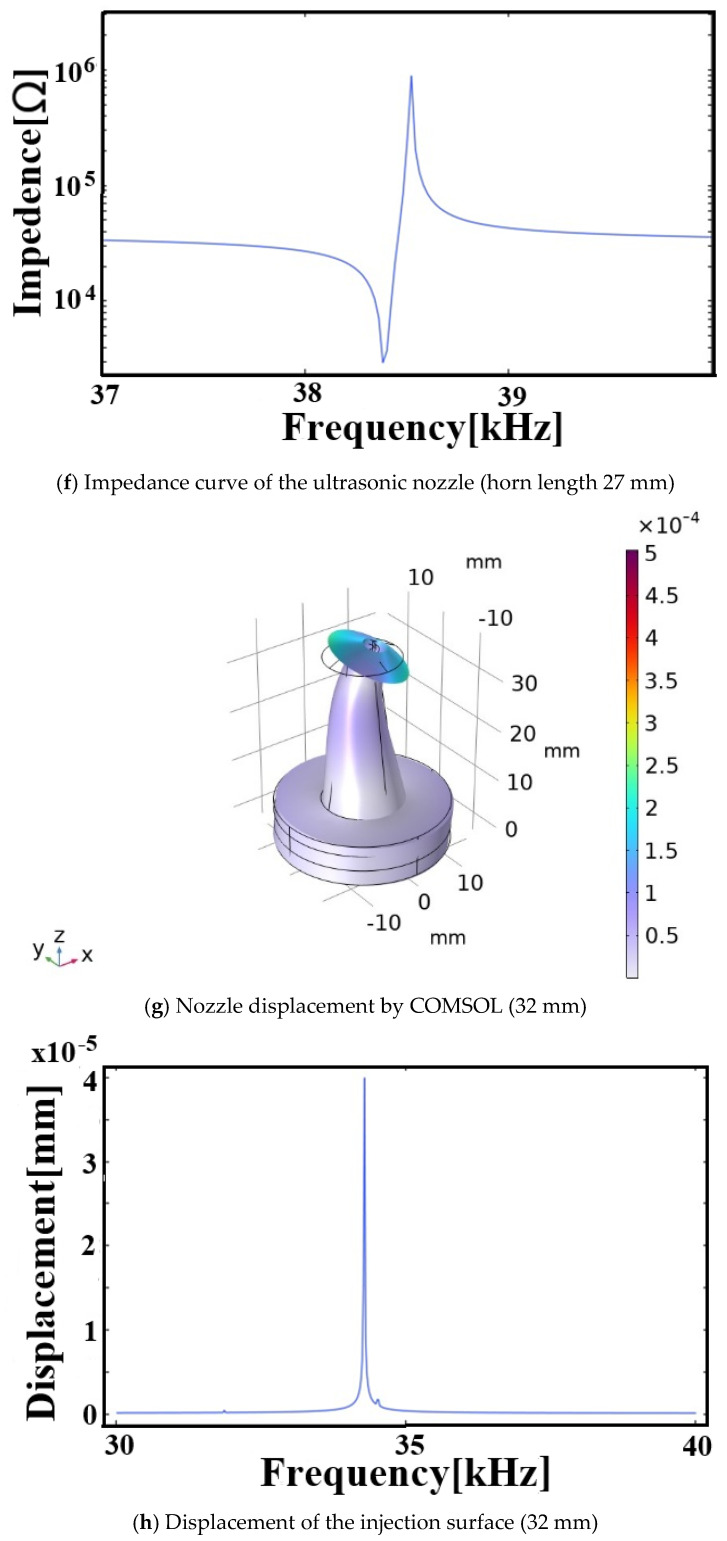

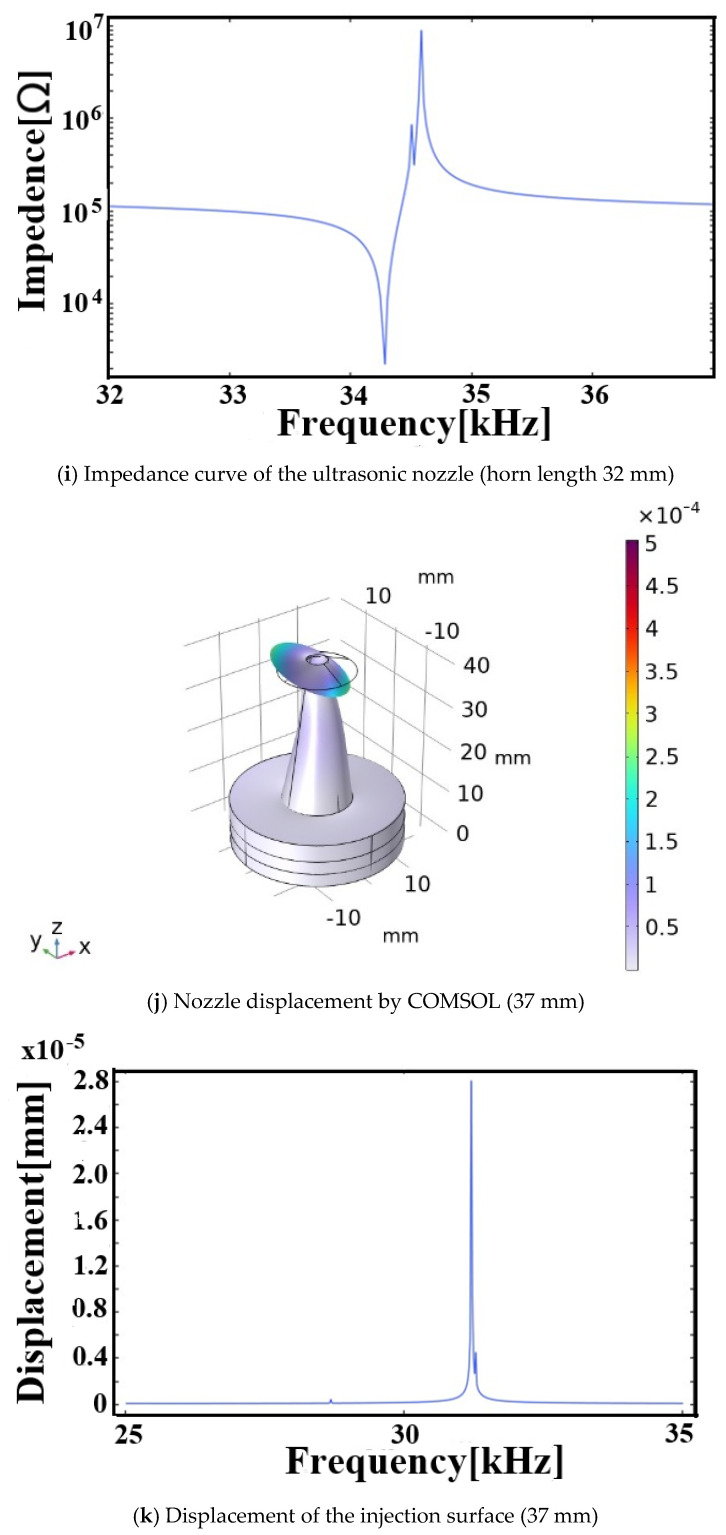

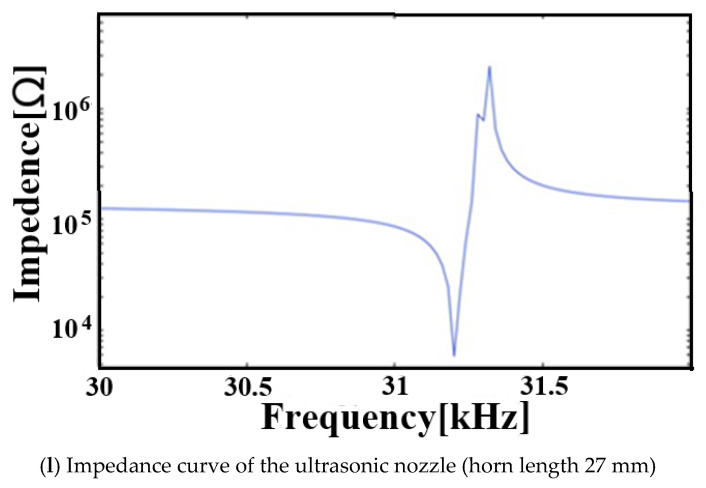
COMSOL analysis of the length of the ultrasonic nozzle based on modeling.

The COMSOL program used in this study was version 6.3, and two modules (COMSOL Multiphysics and Structural Mechanics Module and AC/DC Module) were utilized. To drive the device, the Terminal was set to the side of the disk-type piezoelectric element, an initial value of 1 V was applied, and the Ground was set to the opposite side of the Terminal. A Loss Factor of 0.01 was applied, reflecting the mechanical quality factor (Qm). Additionally, no fixed constraints were set, considering use in the air. Furthermore, the variables used in the simulation were density, Young’s modulus, and Poisson ratio for SUS304, and density, piezoelectric constant (d), elastic flexibility coefficient (s), and relative permittivity (ε) for the ceramic vibrator [30]. Figure 6a shows the displacement of the ultrasonic nozzle at the spray surface, as analyzed using the COMSOL program. The rear of the ceramic vibrator is fixed to the device, so the nozzle vibration is assumed to originate from the ceramic vibrator to the spray surface. In addition, the results obtained through the COMSOL program to determine the degree of displacement according to the length of the horn of the ultrasonic nozzle (22 mm, 27 mm, 32 mm, and 37 mm) are shown in Figure 6. Figure 6a,d,g,j shows that the greatest displacement occurs at the spray surface, regardless of nozzle horn length. The displacement magnitude, calculated using the COMSOL program, is shown in Figure 6b,e,h,k as a function of nozzle horn length. The shorter the ultrasonic nozzle horn length, the larger the resonant frequency, and the higher the resonant frequency, the greater the displacement. It can be predicted that a larger displacement would result in a smaller spray droplet size, which is in good agreement with the theoretical notion that the total energy received by a single liquid particle is proportional to the frequency. In addition, Figure 6c,f,i,l, shows the impedance characteristic curve according to the ultrasonic nozzle horn length obtained using the COMSOL program. It shows the smallest resistance value at the resonant frequency, which also matches well with the resonant frequency of the actually manufactured ultrasonic nozzle. It can be seen that the longer the ultrasonic nozzle, the smaller the resonant frequency. This is in good agreement with the displacement in Figure 6b,e,h,k. Furthermore, the COMSOL program can be used to predict particle size. Furthermore, by studying data on resonant frequency, displacement, liquid type, and flow rate, it is expected that desired particle size control will be possible.

**Figure 7:**
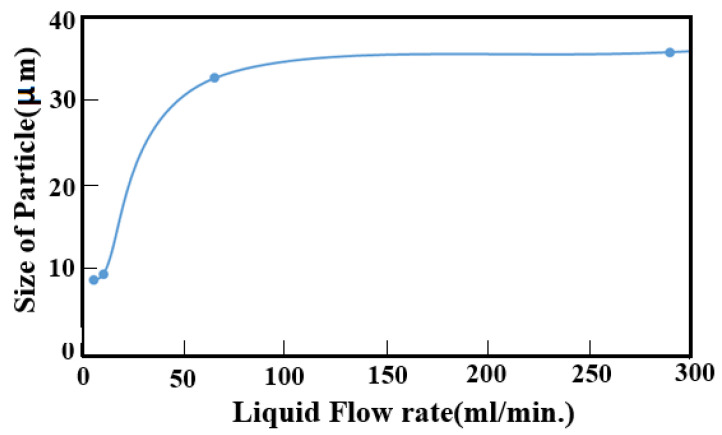
Average size of spray particles according to flow rate change.

Table 2 summarizes the resonance frequency, anti-resonance frequency, resonance resistance values, and maximum displacement obtained through COMSOL analysis. In addition, values obtained by measuring the actually fabricated nozzle are also presented to demonstrate the agreement between the analysis results of the COMSOL program and the experimental values. Figure 7 shows the results of examining the spray particle size according to the change in flow rate ml/min. from an ultrasonic nozzle using pure water. The spray particle size was measured at a distance of 5 mm from the spray inlet using an automatic particle counter (MS-S, UK, Malvern, 2000). In addition, in order to have the same conditions, a nozzle horn length of 22 mm was used and the flow rates were varied to 5, 10, 65, and 290 mL/min. This is because very slow and fast flow rates were selected to examine the change in particle size according to flow rate, and 10 and 65 mL/min. were selected as flow rates that are widely used in industry. The average particle size increased as the flow rate (or flow velocity) increased, which is in good agreement with the result in Equation (8) above, which states that the total amount of energy received by one liquid particle is inversely proportional to the flow rate (or velocity). In addition, the fact that the shorter the nozzle horn length, the higher the resonant frequency, and the total amount of energy received by one liquid particle increases can be explained by Equation (8).

**Figure 8:**
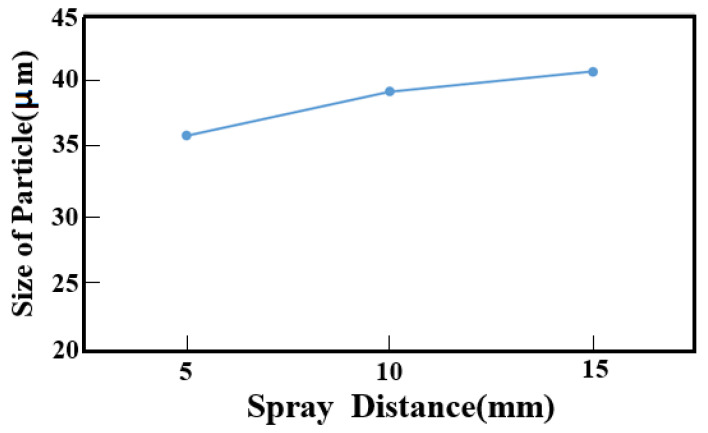
Spray particle size according to spray distance.

Figure 8 shows the size of the spray particles according to the spray distance when spraying 65 mL/min, which is the amount of fuel typically used in small boilers. The size of the spray particles is mostly 30 to 40 μm, showing a relatively uniform particle size distribution, which allows for the expectation of increased combustion efficiency when combusted. In addition, the larger the spray particles, the farther they travel from the nozzle’s spray surface, indicating that the larger the particle size, the greater the mass of the spray particles, allowing for further delivery.

**Figure 9:**
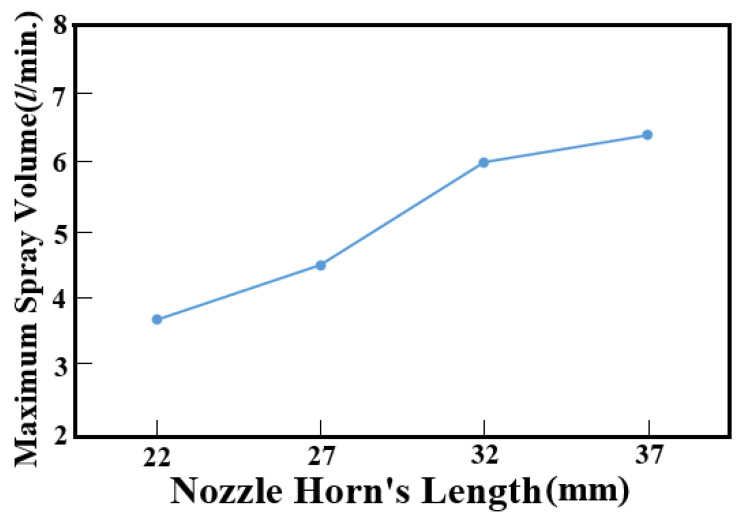
Maximum spray volume according to nozzle horn length.

Figure 9 shows the maximum spray volume according to the ultrasonic nozzle horn length. It can be seen that the maximum spray volume increases as the nozzle horn length increases. This is because as the nozzle horn length increases, the resonant frequency of the nozzle decreases, thereby reducing the energy supplied to the liquid. This is in good agreement with the result of Equation (11). Therefore, in order to reduce the total surface area of the sprayed particles, the diameter of each sprayed particle must be increased. This results in an increase in the spray volume per unit time, as the average size of the sprayed particles must increase.

**Figure 10:**
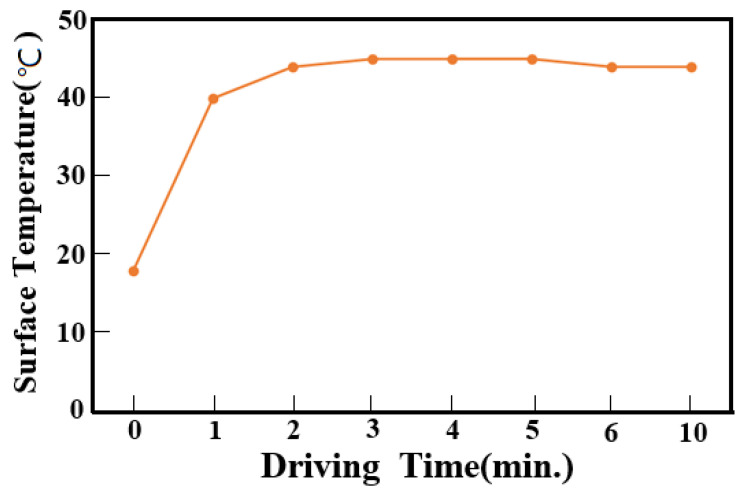
Surface temperature according to the operating time of the ultrasonic nozzle.

In the case of ultrasonic nozzles, the reverse piezoelectric effect of piezoelectric ceramics is utilized, and since the heat generated by mechanical vibration causes depolarization of the ceramic, a composition with a Curie temperature of 300 °C or higher was selected in the ceramic composition for ultrasonic nozzles. Therefore, the temperature generated during nozzle operation is an important factor in nozzle usage. Figure 10 shows the degree of temperature rise in the ultrasonic nozzle during vibration. Although the nozzle surface temperature rose to 45 °C after 3 min of operation, it maintained a stable state of 45 °C thereafter. Therefore, it is expected that ceramic degradation will not occur and energy loss will be reduced.

**Figure 11:**
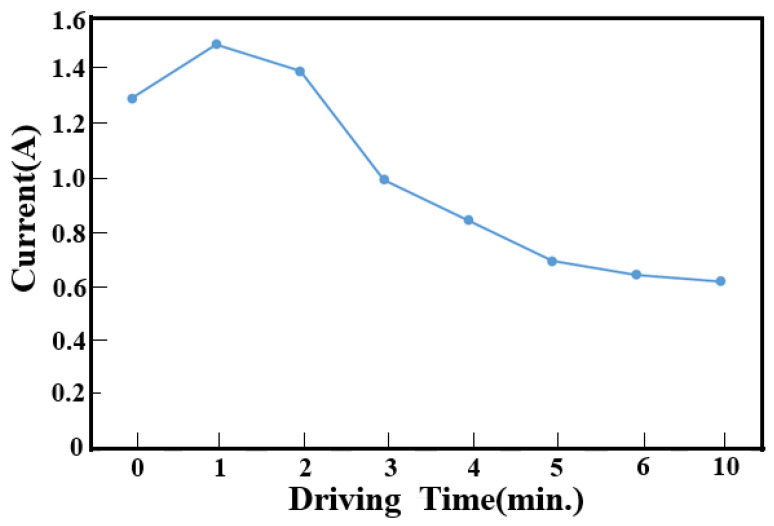
Driving current according to the driving time of the ultrasonic nozzle.

**Figure 12:**
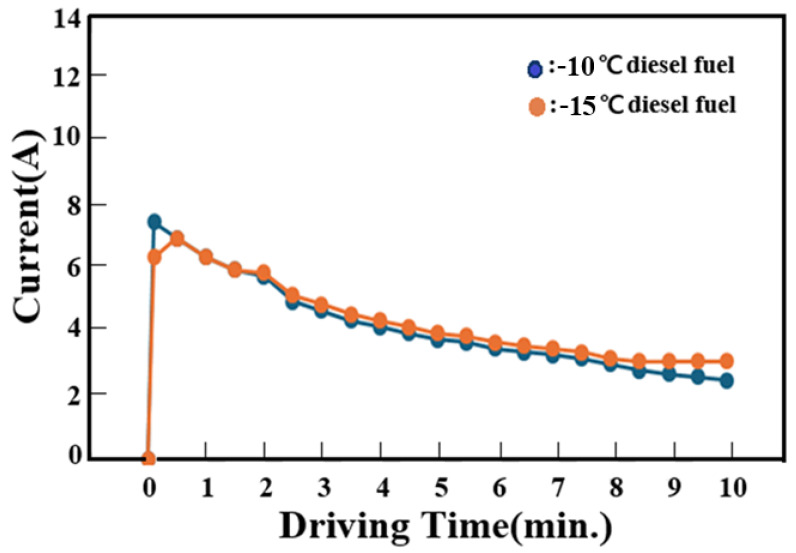
Driving current according to the driving time of the ultrasonic nozzle for diesel fuel.

The heat generated when an ultrasonic nozzle is driven is greatly affected not only by mechanical vibration but also by the value of the current applied to the device. Therefore, Figure 11 shows the driving current according to the driving time when the nozzle horn length is 22 mm and the spray volume of pure water is 10 mL. The voltage supplied at this time is a sine wave frequency of 42.2 kHz with a maximum peak of 360 V. In Figure 11, the driving current requires a high current of 1.5 A during the initial driving, but as it stabilizes, it decreases to about 0.6 A and is supplied constantly, so it can be seen that the surface temperature of the nozzle is stabilized, as seen in the results of Figure 10. Also, since there is a term for the viscosity coefficient of the liquid in Equation (11), the spray characteristics for diesel fuel were examined in Figure 12 to examine the spray characteristics according to the change in viscosity. This was intended to suggest the possibility of industrial application. In the case of diesel fuel, the kinematic viscosity is about 1.9 to 5.5 cSt at room temperature, which is about 3 to 6 times higher than that of pure water. Here, kinematic viscosity is a physical value that indicates natural flow due to gravity, and it refers to the value obtained by dividing the viscosity by the density of the object. In particular, since the viscosity of liquid fuels increases as the temperature decreases, changes in the energy required for viscosity change can be verified through spray tests at low temperatures [−10 °C, −15 °C]. Generally, in the case of diesel fuel used as fuel for diesel engines, a cloud point occurs at 0 °C, causing the wax (paraffin) components of the diesel fuel to appear like a cloud. As crystallization occurs as the temperature decreases, unstable spraying occurred in this study below −15 °C due to crystal growth, resulting in a lack of continuous spraying. Compared to the case of pure water in Figure 11, a high spray driving current of about 8 A is required, and even when the spray is stabilized, it can be seen that the electrical energy required is about 2.5 A. This was intended to suggest the possibility of using the manufactured ultrasonic nozzle in winter, as most industrial applications using oil are used in winter.

## 4. Conclusions

This paper presents a theory of atomization for ultrasonic nozzles, utilizing the vibration characteristics of piezoelectric ceramics to atomize flowing fluids. The theoretical modeling was then experimentally validated for variations in various factors. Furthermore, the displacement of the ultrasonic nozzle was analyzed using COMSOL modeling. The modeling yielded a nozzle that exhibited maximum displacement, demonstrating the appropriateness of the modeling. The results of this study are summarized below.

1. Among the ceramics substituted with 0, 3, 5, 7, and 9 mol% of PMS, the ceramic substituted with 3 mol% exhibited suitable piezoelectric properties as an ultrasonic transducer (mechanical quality factor 1003, piezoelectric constant 308 pC/N, electromechanical coupling coefficient 0.584). In addition, a high Curie temperature of 308 °C, which is required for ultrasonic transducers that require stability at high temperatures, was observed.
2. Through modeling, an ultrasonic nozzle was designed that generated a maximum displacement of 0.48 μm when the nozzle horn length was 22 mm. A larger displacement resulted in maximum energy transfer, allowing for fine-tuning of the spray particle size.
3. A longer ultrasonic nozzle horn resulted in a lower resonant frequency, reducing the energy transferred to each liquid particle and increasing the average spray particle size. However, the maximum spray volume increased due to the larger particle size of the separated particles.
4. It was found that for an ultrasonic nozzle with a nozzle horn length of 22 mm at a flow rate of 65 mL/min, the average particle size at a spray distance of 5 mm was 30–40 μm, and the maximum spray volume was 3.7 L/min.
5. To verify the feasibility in actual industrial applications, a spray test was conducted on high-viscosity diesel fuel, and it was found that higher energy is required for spraying than with pure water. In addition, it was confirmed that spraying is possible through spray tests at low temperatures [−10 °C, −15 °C] where the kinematic viscosity increases further to 15 cSt or higher. Therefore, this suggests that industrial application is possible as a spraying device for diesel fuel, which is widely used in winter.

This study aims to design an ultrasonic nozzle that controls the uniform atomization particle size of diesel fuel particles in order to develop a high-efficiency industrial combustion boiler capable of contributing to the resolution of recent environmental and energy issues. Additionally, we plan to conduct further research on efficiency by burning actual atomized diesel fuel. To this end, we aim to design and fabricate a nozzle capable of generating maximum heat output through systematic combustion tests and efficiency calculations based on particle size control and to apply it to an actual industrial boiler.

## Figures and Tables

**Table 1:** Characteristics of piezoelectric ceramics according to PMS substitution amount.

PMS Substitution	Density [g/cm^3^]	Dielectric Constant [ε_r_]	Electromechanical Coupling Factor [k_p_]	Mechanical Quality Factor [Q_m_]	d_33_ [pC/N]
0 [mol%]	7.882	1271	0.394	396	250
3 [mol%]	7.926	1005	0.584	1003	308
5 [mol%]	7.928	844	0.551	823	274
7 [mol%]	7.952	837	0.507	668	239
9 [mol%]	7.949	935	0.434	591	201
PZT series	7.6~7.7	1000~1100	0.4~0.6	500~2000	200~300

**Table 2:** Resonance characteristics via COMSOL analysis.

Horn Length [mm]	fr [kHz]		fa [kHz]		Zr [Ω]		Max Displacement (mm)
	COMSOL Simulation	Experimental Value	COMSOL Simulation	Experimental Value	COMSOL Simulation	Experimental Value	COMSOL Simulation
22	42.22	42.4	42.88	43.3	605	252	5.037 × 10^−4^
27	38.38	38.5	38.52	38.95	291	160	3.082 × 10^−4^
32	34.28	34.4	34.58	34.9	220	334	3.990 × 10^−5^
37	31.2	31.52	31.32	31.9	580	421	2.802 × 10^−5^

## Data Availability

The original contributions presented in this study are included in the article. Further inquiries can be directed to the corresponding author.

## References

[1] Xiong J., Cai J., Kang Y., Wang X., La Q., Li D. (2024). Generation of effective pulsed waterjets by ultrasonic nozzle used for energy exploration. Energy.

[2] Cai J., Xiong J., Wang L., Li D., Kang Y., Ma Y., Chen Y. (2025). Erosion characteristics of pulsed waterjets issuing from a novel ultrasonic nozzle. Phys. Fluids.

[3] Hou W., Fan S., Li X., Guo X., Fang Z. (2025). Mode decomposition of pressure effects on coherent structures in self-excited oscillating cavitation waterjets. Phys. Fluids.

[4] Ge J., Lin Y., Qi H., Li Y., Li X. (2024). The impact of ultrasonic-induced jet morphology on polishing efficiency. Int. J. Mech. Sci..

[5] Jedelsky J., Jicha M. (2013). Energy conversion during effervescent atomization. Fuel.

[6] Wang W., Chen Z., Hasegawa H., Hirano K., Imashiro C., Morita T. (2025). Effect of driving frequency and power on droplet size atomized by a multimodal transducer. Ultrason. Sonochem..

[7] Bóta A., Amenitsch H., Wacha A. (2025). Lamellarity of ultrasound assisted formations of dipalmitoyl-lecithin vesicles. Ultrason. Sonochem..

[8] Tong Z., Wu Z., Gu Y.A., Lou L. (2024). Effect of humid environment on electromechanical performance of piezoelectric micromachined ultrasonic transducers (PMUTs). Sens. Actuators A Phys..

[9] Ramisetty K.A., Pandit A.B., Gogate P.R. (2013). Investigations into ultrasound induced atomization. Ultrason. Sonochem..

[10] Haertiong G.H. (1999). Ferroelectric ceramics: History and technology. J. Am. Ceram. Soc..

[11] Merouani S., Hamdaoui O., Rezgui Y., Guemini M. (2014). Theoretical estimation of the temperature and pressure within collapsing acoustical bubbles. Ultrason. Sonochem..

[12] Klima J., Frias-Ferrer A., Gonzalez-Garcia J., Ludvik J., Saez V., Iniesta J. (2007). Optimisation of 20 kHz sonoreactor geometry on the basis of numerical simulation of local ultrasonic intensity and qualitative comparison with experimental results. Ultrason. Sonochem..

[13] Duan Y., Wang S., Zhang X., Zhang H., Wang H., Chen S. (2025). Mechanistic insight into the frequency-dependent ultrasound-assisted extraction of *Rosa laevigata* Polysaccharides: Structure, antioxidant activity, and process optimization. Ultrason. Sonochem..

[14] Kewalramani J.A., de Souza B.B., Marsh R.W., Meegoda J.N. (2023). Contributions of reactor geometry and ultrasound frequency on the efficiency of sonochemical reactor. Ultrason. Sonochem..

[15] Sun W.J., Zhang J.Z., Ge S.Y., Shi P., Li L.L., Zhang Y., Yuan H., Gui D., Shi Z. (2023). Effect of solid content on the piezoelectric properties of porous (Ba_0.85_Ca_0.15_)(Zr_0.1_Ti_0._9)O_3_ ceramics. Ceram. Int..

[16] Du L., Du X., Zhang L., An Q., Ma W., Ran H., Du H. (2018). Effect of closed pores on dielectric properties of 0.8Na_0.5_Bi_0.5_TiO_3_-0.2K_0.5_Bi_0.5_TiO_3_ porous ceramics. J. Eur. Ceram. Soc..

[17] Yap E.W., Glaum J., Oddershede J., Daniels J.E. (2018). Effect of porosity on the ferroelectric and piezoelectric properties of (Ba_0.85_Ca_0.15_)(Zr_0.1_Ti_0.9_)O_3_ piezoelectric ceramics. Scr. Mater..

[18] Xu J., Shao Y., Feng X., Zhang X., Li H., Yang J., Gao F. (2015). Low sintering shrinkage porous ceramics: Principles, progress. and perspectives. J. Adv. Ceram..

[19] Lu M., Xu X., Fang Y., Xu X., Feng X., Xu H., Xu J., Gao F. (2025). Enhanced piezoelectric properties and electrical resistivity of Na_+_/Nd_3+_ co-doped Ca1−_x_(Na_1/2_Nd_1/2_)_x_Bi_4_Ti_4_O_15_ ceramics for high-temperature applications. J. Mater. Chem. C.

[20] Su F.-C., Guo X.-B., Lu X.-L., Su Z., Qiu W.-H., Tang X.-G., Li S.-F., Li W.-H. (2023). The effects of microstructure on the dielectric, ferroelectric and impedance properties of 0.5Ba(Zr_0.2_Ti_0.8_)O_3_-0.5(Ba_0.7_Ca_0.3_)TiO_3_ ceramics. Phys. B Condens. Matter.

[21] Lin L., Wang H., Xia C., Xu J., Meng X., Yang R., Hu G., Qu Y., Gao F. (2023). Low sintering shrinkage porous mullite ceramics with high strength and low thermal conductivity via foamgelcasting. J. Am. Ceram. Soc..

[22] Lee S.H., Yoo J.H., Yoo K.J., Lee H.S., Chung K.H. (2005). Dielectric and piezoelectric properties of low temperature sintering Pb(Mg_1/2_W_1/2_)O_3_-Pb(Ni_1/3_Nb_2/3_)O_3_-Pb(ZrTi)O_3_ ceramics for multilayer piezoelectric actuator. Jpn. J. Appl. Phys..

[23] Yang Z., Zong X., Chang H.L.Y. (2005). Structure and electrical properties of new Pb(Zr,Ti)O_3_-Pb(Fe_2/3_W_1/3_)O_3_-Pb(Mn_1/3_Nb_2/3_)O_3_ ceramics. J. Mater. Lett..

[24] Asakura Y., Yasuda K. (2021). Frequency and power dependence of the sonochemical reaction. Ultrason. Sonochem..

[25] Qiao P., Yang Y., Wang Y., Zhang J., Wu J., Zhao L., Liu J., Liu H., Liu H., Tang W. (2024). Enhancing the piezoelectric performance of novel PZT-PMS-PSN piezoelectric ceramics by fine-tuning the monoclinic phase. Ceram. Int..

[26] Ma Y., Zhao M., Zhang D., Li Z., Zhang M., Jin L., Yan Y. (2023). Enhanced piezoelectricity in Pb(Zr_0.48_Ti_0.52_)O_3_-Pb(Mn_1/3_Sb_2/3_)O_3_-Pb(Mg_1/3_Ta_2/3_)O_3_ ceramics through the synergistic effect of defect engineering and lattice cistortion. J. Alloys Compd..

[27] Yang Z., Li H., Zong X., Chang Y. (2006). Structure and electrical properties of PZT–PMS–PZN piezoelectric ceramics. J. Eur. Ceram. Soc..

[28] Kauer M., Belova-Magri V., Cairós C., Schreier H.-J., Mettin R. (2017). Visualization and optimization of cavitation activity at a solid surface in high frequency ultrasound fields. Ultrason. Sonochem..

[29] Leong T., Coventry M., Swiergon P., Knoerzer K., Juliano P. (2015). Ultrasound pressure distributions generated by high frequency transducers in large reactors. Ultrason. Sonochem..

[30] Kwak M.S., Hwang G.T. (2021). Structural Analysis Simulation of Cantilever Shaped Piezoelectric Energy Harvester Using COMSOL Multiphysics. J. Electron. Mater..

